# Travel to School and Physical Activity Levels in 9–10 Year-Old UK Children of Different Ethnic Origin; Child Heart and Health Study in England (CHASE)

**DOI:** 10.1371/journal.pone.0030932

**Published:** 2012-02-03

**Authors:** Christopher G. Owen, Claire M. Nightingale, Alicja R. Rudnicka, Esther M. F. van Sluijs, Ulf Ekelund, Derek G. Cook, Peter H. Whincup

**Affiliations:** 1 Division of Population Health Sciences and Education, St George's, University of London, London, United Kingdom; 2 MRC Epidemiology Unit and Centre of Excellence in Diet and Activity Research (CEDAR), Institute of Metabolic Science, Addenbrooke's Hospital, Cambridge, United Kingdom; IUMSP, CHUV/University of Lausanne, Switzerland

## Abstract

**Background:**

Travel to school may offer a convenient way to increase physical activity levels in childhood. We examined the association between method of travel to school and physical activity levels in urban multi-ethnic children.

**Methods and Findings:**

2035 children (aged 9–10 years in 2006–7) provided data on their usual method of travel to school and wore an Actigraph-GT1M activity monitor during waking hours. Associations between method of travel and mean level of physical activity (counts per minute [CPM], steps, time spent in light, moderate or vigorous activity per day) were examined in models adjusted for confounding variables. 1393 children (69%) walked or cycled to school; 161 (8%) used public transport and 481 (24%) travelled by car. White European children were more likely to walk/cycle, black African Caribbeans to travel by public transport and South Asian children to travel by car. Children travelling by car spent less time in moderate to vigorous physical activity (−7 mins, 95%CI-9,-5), and had lower CPM (−32 CPM, 95%CI-44,-19) and steps per day (−813 steps, 95%CI,-1043,-582) than walkers/cyclists. Pupils travelling by public transport had similar activity levels to walkers/cyclists. Lower physical activity levels amongst car travellers' were especially marked at travelling times (school days between 8–9 am, 3–5 pm), but were also evident on weekdays at other times and at weekends; they did not differ by gender or ethnic group.

**Conclusion:**

Active travel to school is associated with higher levels of objectively measured physical activity, particularly during periods of travel but also at other times. If children travelling by car were to achieve physical activity levels (steps) similar to children using active travel, they would increase their physical activity levels by 9%. However, the population increase would be a modest 2%, because of the low proportion of car travellers in this urban population.

## Introduction

Low levels of physical activity in childhood are a major public health concern [1]. The results of recent studies using objective measurements suggest that physical activity levels in UK children are low [2], , and markedly lower than levels measured in children of a similar age in other European countries [4]–[6]. Fewer than two-thirds of children report achieving recommended levels of physical activity of an hour or more of moderate activity per day [1]. Physical inactivity in childhood has adverse consequences for adiposity and cardiometabolic risk factors in childhood [3], [7]–[9]. The need to increase levels of physical activity in children is now recognized in current health policies [1], [10,10]. However, interventions to promote physical activity in young people have so far failed to show consistently beneficial effects [11]; where effects have been demonstrated these have proved difficult to maintain in the longer term [12], [13]. School based interventions offer an opportunity to increase levels of physical activity and reduce sedentary behaviour, although evidence of effectiveness has been mixed [13]–[16]. Travelling to school using active methods (walking or cycling, in combination with public transport where necessary) may provide a convenient way of increasing daily levels of physical activity, which can be integrated into children's lives [17]. However, the proportion of children using active methods of travelling to school have become less common in recent years, with a higher proportion of journeys being undertaken by car [18]–[22]. There is uncertainty as to whether active travel to school confers beneficial effects on overall levels of physical activity in childhood, with studies showing beneficial [23]–[26] or little effect [27]. Previous studies have been in predominantly white, often non-metropolitan populations [23], [24], [26]. Little is known about the impact of active travel to school in multi-ethnic urban populations, especially amongst South Asians who have particularly low levels of physical activity in childhood [2] and adverse patterns of adiposity and cardiometabolic risk [28], [29].

We therefore studied the associations between mode of travel to school and levels of physical activity in UK children of white European, South Asian and black African-Caribbean origin. We also: (i) quantified the impact of changing from car travel to more active forms of travelling to school (walking/cycling/public transport) on levels of physical activity both in the affected children and in the whole population; and (ii) examined whether active travel to school is associated with higher levels of physical activity outside school commuting hours, to gauge whether any difference is part of a more general difference in lifestyle.

## Methods

### Ethics Statement

Ethical approval for the study was obtained from the Wales Multi-Centre Research Ethics Committee (reference M-07/MRE09/31).

The Child Heart And Health Study in England (CHASE) examined the cardiovascular health of more than 5000 UK children aged 9 to 10 years in 200 primary schools in London, Birmingham and Leicester sampled to provide similar numbers of children of white European, South Asian and black African-Caribbean origin between 2004 and 2007. Levels of physical activity were measured in 2035 of these children in the last 78 schools studied during 2006 and 2007. Full details of the main study and the physical activity study have been provided elsewhere [2]. Invitation letters were sent to parents or guardians of pupils in year 5 classes; translations were provided where necessary. Written informed consent was obtained from all parents or guardians. Measurements were made by a trained field team who visited schools in North-West London, North-East London and South London on a fortnightly schedule, with periodic visits to Birmingham and Leicester.

### Physical activity assessment

Children were asked to wear an Actigraph GT1M activity monitor (ActiGraph, LLC, Pensacola, FL, USA), during waking hours for 7 whole days. The monitor, programmed to record at 5 second epochs, was positioned over the left hip and maintained in position with an elasticised belt. A gift voucher was issued on safe return of the monitor to school on the eighth day (the following Monday if this fell on a weekend). ActiGraph data files were downloaded (omitting the first and last incomplete days) and batch processed using a dedicated programme (MAHUFFE available from http://www.mrc-epid.cam.ac.uk/Research/Programmes/Programme_5/InDepth/Programme%205_Downloads.html). Activity outcomes included mean daily activity counts, mean daily steps, and activity counts per minute (CPM) of registered time. Registered time was defined as the total period accepted for analysis (with time periods of at least 20 consecutive minutes of zero counts being excluded as periods of non-wear). Days with at least 600 minutes of registered time were included for analysis; no limitation was placed on the number of days with a sufficient duration of recording. Mean daily time spent in sedentary (<100 CPM), light (100 to <2000 CPM), moderate 2000 to <4000 CPM) and vigorous (≥4000 CPM) levels of activity was identified. A category defined as moderate to vigorous physical activity (MVPA) was also used by combing the latter two levels. The threshold for moderate activity is equivalent to walking 4 km per hour in children, which will be the predominant form of active transport [30]–[32]. Hence, we did not apply higher thresholds which have been used previously to define moderate activity (3600 CPM) in a similar age group [33]. Levels of physical activity during weekdays were also examined by the hour (integer units only), comparing periods of travel (between 8 to 9 am and 3 to 5 pm) with the remainder of the day.

### Distance from home to school, ethnicity and parental social class

Mode of travel to school was ascertained from child questionnaires. Children were asked ‘How do you usually travel to school?’ and given the option to respond ‘By car’, ‘By bicycle’, ‘By bus or train’ or ‘Walking’. Responses were classified as (i) walking/cycling, (ii) public transport – bus/train, and (iii) car. These categories were chosen a priori. Distance from home to school was calculated as the Euclidean distance between home and school postcodes [34]. The ethnic origin of the child was based on parental information on the self-defined ethnicity of both parents, or (where not available) parentally defined ethnicity of the child. In a small number of children where this information was not available (n = 20), ethnic origin was based on information provided by the child on parental and grand-parental place of birth. Children of unmixed ethnic origin were classified as white European, South Asian, and black African-Caribbean. Children of other ethnic origins and of mixed ethnic origin were allocated to a separate ‘other ethnic groups’ category. Information on parental occupation was collected from the parents or (if not available) from the child and was used to code social class using SOC-2000 classification [35].

### Statistical analysis

Statistical analyses were carried out using STATA/SE software (Stata/SE 10 for Windows, StataCorp LP, College Station, TX, USA). Outcome variables included mean daily counts, steps, CPM, and time spent in different levels of activity. All activity outcomes appeared normally distributed. Multilevel linear regression models taking account of the natural clustering of children within school and repeated days within individuals were used to provide adjusted means and mean differences in levels of physical activity by mode of travel to school (walking/cycling, public transport, or car) for (i) weekdays and (ii) weekends. Most children walked or cycled, and hence these were used as the reference group. Hourly data were used to ascertain the level of physical activity carried out during periods of travel to school (defined as 8 to 9 am, and 3 to 5 pm on a weekday). Physical activity levels outside weekday periods of travel were also examined. Plots of distance of travel from home to school by activity outcome were used to examine patterns of physical activity amongst children who walked/cycled, used public transport, or were driven by car, during travel periods. Tests for interaction did not provide evidence of difference in association between mode of travel to school and levels of activity by gender (all P-values>0.05), or ethnic group (all P-values>0.05). Hence, all analyses were adjusted for age in quartiles, gender, ethnic group, month, day of the week (to allow for higher levels on weekdays compared to weekends), and day order of recording (to allow for higher levels of physical activity on earlier days or recording, despite omission of the first day) [2], [7]. Day of the week and day order were adjusted for in both weekday and weekend analyses. Additional adjustment for socioeconomic position was also examined [35]. We estimated the potential effect on physical activity of changing transport mode from car use to active transport mode by adding the difference in step count between active transport and car use children to the values in car users. The impact on physical activity levels was examined both for car users alone and for the whole study population.

## Results

Of 3449 children invited, 2144 (62%) took part in the Actigraph physical activity survey. Among these, 2071 recorded >600 min of registered time on at least one day, 1841 (89%) on at least 3 days and 1401 (68%) on at least 5 days. The demographic, ethnic and anthropometric characteristics of study participants who wore or did not wear an Actigraph were similar. The mean age of participants was 9.9 years (SD 0.4 years, age quartiles; ≤9.67 years, >9.67 to 9.95 years, >9.95 to 10.21, >10.21 years); 48% were boys. Information on mode of travel to school was provided by 2035 of these 2071 children, with similar response rates (64% South Asian, 59% black African-Caribbean , 63% white European) and numbers of participants by ethnic group (481, 564, 501 respectively). Most children (68.5%) either walked or cycled to school (of which only 15 children [1%] cycled); 23.6% travelled by car, 7.9% used public transport. There were an insufficient number of cyclists for them to be treated as a separate group; exclusion of cyclists had little impact on the findings throughout. Factors related to mode of travel are shown in Table 1. Although mode of travel was unrelated to gender, it was strongly related to ethnicity. White European children were more likely to walk or cycle to school, black African Caribbeans to travel by public transport, and South Asians to travel by car. Mode of travel to school was also strongly related to the distance between home and school. Those living furthest from their school (>0.5 miles) were more likely to travel by car, while those living closest (<0.3 miles) were more likely to walk or cycle to and from school (Table 1). South Asian children tended to live closer to school compared to white European and Black African Caribbean children. White Europeans lived a median distance of 0.4 miles (inter quartile range [IQR] 0.2, 0.7) from school, South Asians 0.3 miles (IQR CI 0.1, 0.4), black African Caribbeans 0.4 miles (IQR 0.2, 0.8). Thus South Asians lived closest to school, but were most likely to travel by car.

**Table 1:** Mode of transport to school by gender, ethnic group, and distance from home to school.

	Walking/Cycling	Public Transport (bus/train)	Car	All modes of Transport
**All (row %s)**	1393 (68.5%)	161 (7.9%)	481 (23.6%)	2035 (100.0%)
**Gender (col %s)**				
Boys	652 (46.8%)	78 (48.4%)	244 (50.7%)	974 (47.9%)
Girls	741 (53.2%)	83 (51.6%)	237 (49.3%)	1061 (52.1%)
P-value †			0.33	
**Ethnic group (col %s)** §				
WE	361 (34.0%)	28 (22.4%)	112 (31.2%)	501 (32.4%)
SA	343 (32.3%)	11 (8.8%)	127 (35.4%)	481 (31.1%)
BAC	358 (33.7%)	86 (68.8%)	120 (33.4%)	564 (36.5%)
P-value †			<0.0001	
**Distance from home to school (miles)** * **(col %s)**				
0–0.2	703 (51.4%)	1 (0.6%)	54 (11.4%)	758 (37.9%)
>0.2–0.5	466 (34.0%)	23 (14.6%)	124 (26.3%)	613 (30.7%)
>0.5	200 (14.6%)	134 (84.8%)	294 (62.3%)	628 (31.4%)
P-value ‡			<0.0001	

WE = white Europeans, SA = South Asians, BAC = black African Caribbeans, row %s = row percentages, col %s = column percentages.

† Pearson Chi^2^ test for difference between categories.

‡ Fisher's Exact test for difference between categories.

§ ‘Other’ ethnic group has been removed from the ethnic group analysis.

*Distance to school in tertiles – number of subjects with missing data = 36.

The relations between travel mode and physical activity on weekdays (i.e. school days) are presented in Table 2. Compared to children who walked or cycled to school, weekday activity counts (counts, CPM, steps) were lower amongst children who travelled to school by car (Table 2). Children who travelled by car also spent fewer minutes in moderate or higher levels of activity than those who walked or cycled (Table 2). Children who used public transport had similar weekday activity counts and CPM to those who walked or cycled but accumulated more steps. They also spent longer in moderate and MVPA than those who walked or cycled (Table 2).

**Table 2:**
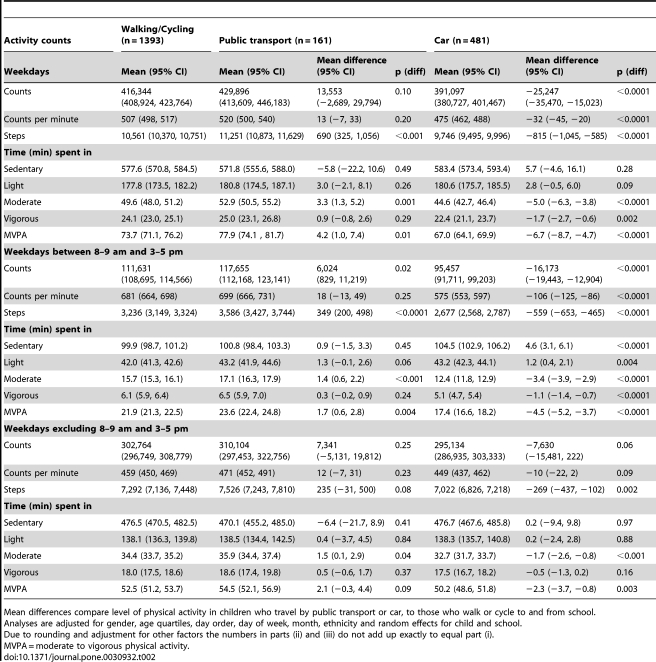
Adjusted mean weekday levels of physical activity by mode of transport to school (i) on weekdays, (ii) between 8 to 9 am and 3 to 5 pm on weekdays, (iii) on weekdays excluding periods of active travel.

Mean differences compare level of physical activity in children who travel by public transport or car, to those who walk or cycle to and from school.

Analyses are adjusted for gender, age quartiles, day order, day of week, month, ethnicity and random effects for child and school.

Due to rounding and adjustment for other factors the numbers in parts (ii) and (iii) do not add up exactly to equal part (i).

MVPA = moderate to vigorous physical activity.

**Figure 1:**
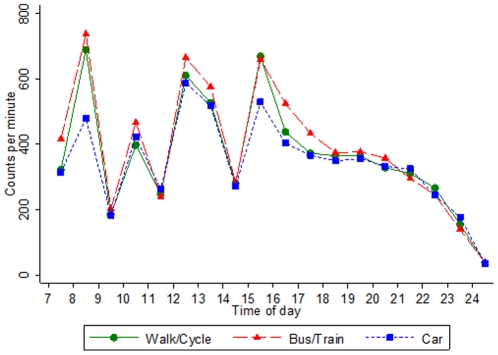
Median weekday physical activity levels (CPM) from 7 am to midnight by mode of travel to school.

We examined the differences in physical activity patterns between travel modes separately during periods of travel to school, in other weekday periods (Table 2), and at weekends (Table 3). The lower hourly rates of activity counts, CPM and steps observed among those travelling by car compared with those walking or cycling were particularly marked, and time spent in higher levels of activity shorter, during periods of travel to school. However, at other weekday periods excluding travel times, those travelling by car still had lower step counts, and spent less time in moderate and MVPA (Table 2). At weekends an almost identical pattern to the overall weekday pattern was apparent, with lower counts, CPM and steps and shorter periods spent in moderate and MVPA seen in the car travelling group (Table 3). During weekday periods of travel, children using public transport recorded similar CPM but had higher hourly rates of counts and steps and longer durations of moderate and MVPA compared to children walking or cycling (Table 2). However, in other weekday periods excluding travel times, children using public transport generally had similar levels of physical activity to those walking or cycling, except for a slightly higher duration of time spent in moderate activity (Table 2). At weekends, children using public transport had similar levels of physical activity to walkers and cyclists (Table 3). Hourly levels of weekday CPM from 7 am to midnight are summarised for the three travel modes in Figure 1. Lower levels of physical activity among car travellers were apparent during commuting times and during the lunch hour compared to those using active forms of travel; similar differences were observed in the total number of counts and steps (Supplemental Figures 1 and 2). There was no evidence to suggest that these associations between mode of travel and physical activity differed between males and females or by ethnic group (all tests for interaction P>0.05, data not presented). Adjustment for socioeconomic position had little impact on the findings.

**Figure 2:**
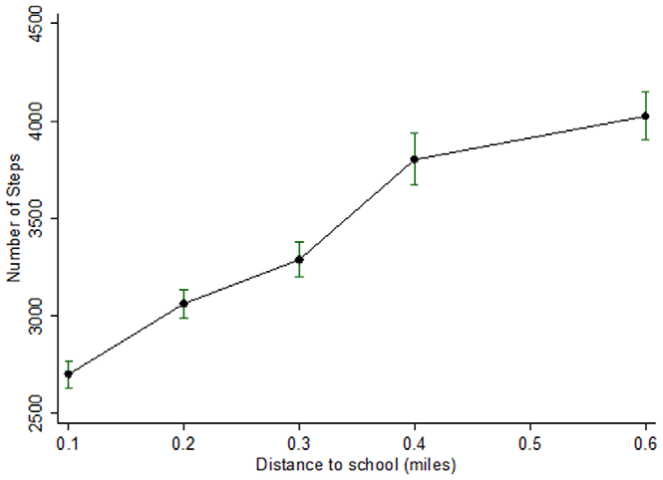
Mean (95% CI) weekday physical activity levels (steps) by median distance to school between 8 to 9 am and 3 to 5 pm on weekdays in walkers only (no other forms of transport used).

**Table 3:** Mean weekend levels of physical activity by mode of transport to school.

Activity counts	Walking/Cycling (n = 1393)	Public transport (n = 161)			Car (n = 481)		
	Mean(95% CI)	Mean (95% CI)	Mean difference (95% CI)	p (diff)	Mean (95% CI)	Mean difference (95% CI)	p (diff)
Counts	356,016 (346,628 , 365,405)	367,717 (344,464, 390,969)	11,700 (−12,122, 35,523)	0.34	336,762 (322,775, 350,748)	−19,255 (−34,008, −4,502)	0.01
Counts per minute	450 (439, 462)	466 (437, 495)	15 (−15, 45)	0.32	430 (413, 448)	−20 (−38, −1)	0.04
Steps	8,218 (8,009, 8,428)	8,343 (7,781, 8,904)	124 (−459, 708)	0.68	7,408 (7,079, 7,736)	−811 (−1,172, −450)	<0.0001
**Time (min) spent in**							
Sedentary	567.8 (558.4, 577.3)	562.0 (537.7, 586.4)	−5.8 (−30.9, 19.3)	0.65	564.5 (550.1, 578.9)	−3.3 (−18.8, 12.2)	0.68
Light	173.2 (168.7, 177.7)	171.2 (162.8, 179.6)	−2.0 (−9.9, 5.9)	0.62	170.2 (164.5, 175.9)	−3.0 (−8.0, 1.9)	0.23
Moderate	42.3 (40.8, 43.8)	44.4 (41.4, 47.5)	2.1 (−0.9, 5.2)	0.16	38.2 (36.2, 40.2)	−4.1 (−6.0, −2.2)	<0.0001
Vigorous	18.2 (17.3, 19.1)	19.7 (17.7, 21.7)	1.4 (−0.6, 3.4)	0.16	17.2 (16.0, 18.5)	−1.0 (−2.3, 0.2)	0.10
MVPA	60.5 (58.3, 62.8)	64.1 (59.4, 68.8)	3.5 (−1.1, 8.2)	0.13	55.4 (52.4, 58.4)	−5.1 (−8.0, −2.3)	<0.001

Mean differences compare level of physical activity in children who travel by public transport or car, to those who walk or cycle to and from school.

Analyses are adjusted for gender, age quartiles, day order, day of week (i.e., Saturday or Sunday), month, ethnicity and random effects for child and school.

Distance from home to school showed a strong positive association with levels of physical activity amongst those who walked/cycled to school, especially during periods of travel (Table 4). A near linear association between distance from home to school and number of steps recorded during periods of travel to school is shown in Figure 2. Distance from home to school showed no consistent pattern with physical activity levels amongst those travelling by car and public transport (data not presented).

**Table 4:** Adjusted activity levels in children who walk/cycle to school by distance to school (i) between 8 to 9 am and 3 to 5 pm on weekdays, (ii) on weekdays excluding periods of active travel.

Distance from home to school (miles) †	Counts per minute (95% CI)	Counts (95% CI)	Steps (95% CI)
**Weekdays 8–9 am and 3–5 pm**			
0–0.2	640 (619, 662)	104,235 (100,658, 107,811)	2,991 (2,889, 3,094)
>0.2–0.5	717 (694, 741)	117,742 (113,820, 121,664)	3,449 (3,337, 3,561)
>0.5	758 (726, 790)	126,386 (121,122, 131,650)	3,738 (3,588, 3,888)
p (linear trend)	<0.0001	<0.0001	<0.0001
**Weekdays excluding 8–9 am and 3–5 pm**			
0–0.2	450 (438, 462)	297,476 (289,437, 305,515)	7,221 (7,023, 7,418)
>0.2–0.5	462 (449, 476)	302,332 (293,417, 311,247)	7,235 (7,021, 7,449)
>0.5	473 (455, 492)	311,173 (298,956, 323,390)	7,380 (7,101, 7,659)
p (linear trend)	0.01	0.03	0.31

† Distances from home to school in tertiles.

Analyses are adjusted for sex, age quartiles, day order, day of week, month, ethnicity, distance to school in tertiles and random effects for child and school.

If children travelling by car were to increase their number of steps on weekdays to a level similar to those using active transport methods (i.e. the average of the walking/cycling and public transport groups, 10,632 steps per weekday) they would increase their steps by approximately 900 steps (9%). However, the proportion of children travelling by car to primary school is modest (24%), and the average weekday increase in population physical activity levels if all car users increased to active forms of travel group would only be from an overall average of 10,423 to 10,632 steps; a change of 209 steps or a 2% increase. Moreover, only about two-thirds of the overall weekday difference in physical activity is directly attributable to active commuting (i.e. it occurs during the 3 school commuting hours), so the direct effect of car users changing to active travel would be a 6% increase in weekday steps for those individuals and a 1.3% increase in steps in the population as a whole. Although the prevalence of car use is slightly higher among South Asians (26%), the potential impact of changing to active travel in this group is not materially different (1.4%). Similar findings are observed if counts or time spent in MVPA are used rather than steps (data not presented).

## Discussion

This study shows that children using active forms of travel to school (walking, cycling, public transport) have higher levels of physical activity than those travelling to school by car. While other studies have shown that active travel is associated with higher weekday levels of physical activity [23], [36]–[38], there is controversy over the contribution these higher levels make to overall levels of physical activity, and whether higher levels are observed outside school commuting hours [39], [40]. The results of a large UK study in predominantly white children aged 11 years suggested that children who walk or cycle to school have higher levels of overall physical activity and spend longer in higher levels of physical activity (approximately 8 minutes more weekday MVPA) compared to those who travel by car [26]. However, higher physical activity levels were not observed at weekends in children who walked or cycled to school [26]. We observed similar weekday findings in our multi-ethnic sample of children of a similar age (32 CPM higher, 7 minutes more MVPA in those who walk/cycle compared with those who travel by car), but we also observed higher levels of activity amongst children using active modes of transport to school at weekends (20 CPM, 5 minutes more MVPA); similar to findings from other smaller studies [36], [41]. In addition, we observed that children using public transport had equivalent or higher levels of physical activity compared to those who walked or cycled to school. We believe this novel finding reflects the amount of walking required to and from public transport embarkation/disembarkation points in this densely populated urban setting. The similarity in physical activity levels between children walking/cycling and using public transport suggests that while children using public transport live further from school, the average distance actually walked by them is similar to those of children who live closer and walk to school. We have examined the behaviour of the activity monitors while using public transport and we are confident that these findings are not explained by artefactual movement recorded while using public transport. However, these public transport findings, based predominantly in London, may not be representative of the experience of children using public transport in other settings and need further replication.

Objective validated assessment of physical activity by means of movement sensors (such as the Actigraph) has allowed better characterisation of the distribution of overall physical activity levels between weekdays and weekends, and of physical activity levels during specific time-periods when higher levels could be achieved. Using activity data from one or more days maximised the study population size, but results were similar when restricted to those with 3 or more days of activity data. In this study, periods of active travel were defined as 8 to 9 am and 3 to 5 pm, which we believe will include the periods of active travel in most if not all children, though in a few it may also include periods of play before or after school (including after school clubs). Hence, this may overestimate the contribution of active travel to overall levels of activity if children who walk/cycle or use public transport are consistently more active during travel periods. A previous study used an earlier cut-off of 4 pm to define the period of active travel [26], but on the basis of our findings this may have underestimated the period of actual travel, particularly for those travelling by bus/train (Figure 1). The use of geographic positioning system (GPS) technology may allow periods of travel to be more accurately defined, along with route travelled, in future studies [42].

Other strengths of the present study include the large sample of primary school aged children, with balanced numbers of children of white European, South Asian and African Caribbean origin. Few studies to date have directly examined the impact of active commuting to school across ethnic groups [39]. Our findings suggest that benefits of active commuting to school are evident in all ethnic groups; South Asians are more likely to benefit from adopting active travel as they live closest to school and are particularly likely to travel to school by car compared to other ethnic groups. However, the differences in active travel do not account for the ethnic differences in physical activity levels, particularly the lower levels in South Asians previously reported [2]. Although most participants contributed 3 or more days of recording, participant inclusion was maximised by including all children with at least one day of physical activity data. This minimised the potential for selection bias, where those with very high or very low levels of physical activity may not participate. A further strength of the study was the objective assessment of distance between home and school, based on Euclidean distance between postcodes. Earlier studies have shown that the difference in physical activity levels between those walking and travelling to school by car becomes greater with increasing distance travelled [26]. We observed similar findings, but we also showed that those travelling by public transport were also more active.

A key question which this and other cross sectional studies are unable to answer is whether active travel promotes a more active lifestyle or is indicative of a more active lifestyle. The higher levels of physical activity at non-commuting times (which account for approximately a third of the differences between those walking/cycling and using car transport) may reflect the fact that children who are a priori more active choose to walk/cycle to school, or that the process of walking/cycling to school leads to an increase in physical activity in non-commuting times. Only longitudinal and interventional studies which assess physical activity levels before active commuting commences will be able to answer this further.

Nevertheless, active travel to school provides a convenient way to maintain or increase levels of physical activity that can easily be integrated into everyday life, without putting pressure on the school curriculum [17]. In the present study, children travelled a median distance of 0.3 miles (mean 0.6 miles) to school and two-thirds of children lived within half a mile of their school, a distance widely considered to be reasonable for walking to school [43]. Nationally, children aged 5 to 10 years travel an average distance of 1.6 miles to school; this increases to 3.4 miles at ages 11–16 years [19]. Hence, on a national basis, a smaller proportion of children live sufficiently close to school to walk. Nationally 50% cycle or walk to school, 4% use public transport, and 43% travel by car at ages 5–10 years [19], whereas our results show a higher proportion walking or cycling to school (68%), with more using public transport (8%) and fewer travelling by car (24%). This suggests that nationally there may be greater potential for encouraging the use of active transport and thus increasing physical activity levels than is the case in the present study population. However, given the greater home-school distances which occur nationally, an increase in use of public transport would need to be an important part of the strategy for encouraging active transport.

While the shorter home-school distance in the present study may largely account for these differences in mode of transport [44], we also observed that more black African Caribbeans use public transport, and more South Asians travel by car despite living within closer proximity to school. Hence, while active forms of travel are already high in these children and scope to increase active travel may be limited, active travel could be promoted in certain groups, particularly South Asians. While weekday levels of physical activity would increase by about 9% among children travelling by car if they were to adopt active modes of transport (including use of public transport), this has only a modest impact on overall physical activity level (2%) because of the low prevalence of car use in our population. Moreover, only two-thirds of this difference can be attributed to periods of travel (1.3%) and lower levels of physical activity previously observed amongst South Asians [2] are not explained by mode of travel to school (data not presented). This suggests that the difference between the car users and active commuters is attributable to more general lifestyle choices; this is further emphasised by the observation that the difference between the groups is also seen at weekends. The wider public health message from these findings, where nationwide levels of car use are higher with greater distances to school (perhaps too far to walk), is the need to encourage greater use of public transport. This may offer an effective strategy to increase physical activity levels, especially if it were translated into a broader change in travel choices.

Concerns over the physical environment, including levels of traffic, poor provision for pedestrians and cyclists, as well as child safety often discourage parents from allowing their child to adopt active forms of travel [45], [46]. These concerns may be heightened in certain ethnic minority groups [47], such as South Asians. However, further information on the determinants of travel mode, and differences in determinants between ethnic groups is needed. Interventions to encourage active travel to school have shown variable effects [48]–[51]. Only one study assessed the change in distance walked, reporting an increase of 574 metres in 10 year olds in Glasgow; this approximates to 1000 steps and is consistent with our study [51].

In conclusion, walking/cycling to school campaigns (as well as use of public transport) have a contribution to make as part of any concerted initiative to increase physical activity levels in children, particularly if these schemes are universally adopted across ethnic groups. Further work looking at the potential benefit of active travel at older ages when children are at secondary school would be especially worthwhile, as the distances travelled are considerably greater.

## Supporting Information

Figure S1 Median weekday physical activity levels (counts) from 7 am to midnight by mode of travel to school
(TIF)

Figure S2 Median weekday physical activity levels (steps) from 7 am to midnight by mode of travel to school
(TIF)
