# Supplementary figures and images for: Travel to School and Physical Activity Levels in 9–10 Year-Old UK Children of Different Ethnic Origin; Child Heart and Health Study in England (CHASE)
Source: PLoS One. 2012 Feb 3;7(2):e30932. doi: 10.1371/journal.pone.0030932 (PMC3272007; doi:10.1371/journal.pone.0030932)

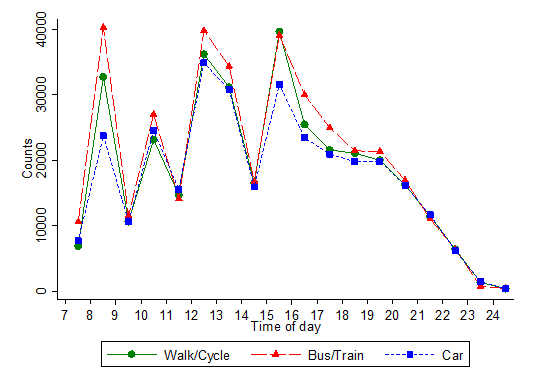

Supplement: Figure S1 — Median weekday physical activity levels (counts) from 7 am to midnight by mode of travel to school (TIF) [file pone.0030932.s001.tif]

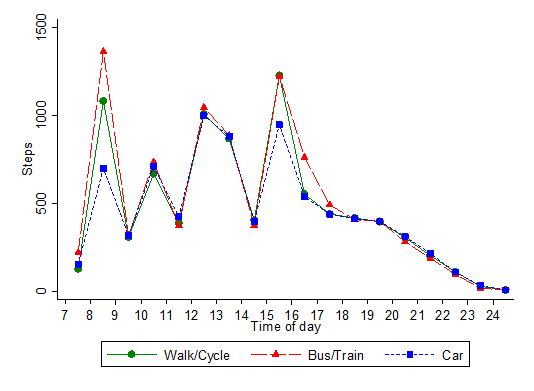

Supplement: Figure S2 — Median weekday physical activity levels (steps) from 7 am to midnight by mode of travel to school (TIF) [file pone.0030932.s002.tif]
